# Metabolomics Profiling for Identification of Novel Potential Markers in Early Prediction of Preeclampsia

**DOI:** 10.1371/journal.pone.0098540

**Published:** 2014-05-29

**Authors:** Sylwia Kuc, Maria P. H. Koster, Jeroen L. A. Pennings, Thomas Hankemeier, Ruud Berger, Amy C. Harms, Adrie D. Dane, Peter C. J. I. Schielen, Gerard H. A. Visser, Rob J. Vreeken

**Affiliations:** 1 Department of Obstetrics, Wilhelmina Children's Hospital, University Medical Centre Utrecht (UMCU), Utrecht, the Netherlands; 2 Laboratory for Health Protection Research (GBO), National Institute for Public Health and the Environment (RIVM), Bilthoven, the Netherlands; 3 Leiden Academic Center for Drug Research, Division of Analytical Biosciences, Leiden University, Leiden, The Netherlands; 4 The Netherlands Metabolomics Centre, Leiden University, Leiden, the Netherlands; 5 Laboratory for Infectious Diseases and Perinatal Screening (LIS), National Institute for Public Health and the Environment (RIVM), Bilthoven, the Netherlands; Instituto de Investigación Sanitaria INCLIVA, Spain

## Abstract

**Objective:**

The first aim was to investigate specific signature patterns of metabolites that are significantly altered in first-trimester serum of women who subsequently developed preeclampsia (PE) compared to healthy pregnancies. The second aim of this study was to examine the predictive performance of the selected metabolites for both early onset [EO-PE] and late onset PE [LO-PE].

**Methods:**

This was a case-control study of maternal serum samples collected between 8+0 and 13+6 weeks of gestation from 167 women who subsequently developed EO-PE n = 68; LO-PE n = 99 and 500 controls with uncomplicated pregnancies. Metabolomics profiling analysis was performed using two methods. One has been optimized to target eicosanoids/oxylipins, which are known inflammation markers and the other targets compounds containing a primary or secondary biogenic amine group. Logistic regression analyses were performed to predict the development of PE using metabolites alone and in combination with first trimester mean arterial pressure (MAP) measurements.

**Results:**

Two metabolites were significantly different between EO-PE and controls (taurine and asparagine) and one in case of LO-PE (glycylglycine). Taurine appeared the most discriminative biomarker and in combination with MAP predicted EO-PE with a detection rate (DR) of 55%, at a false-positive rate (FPR) of 10%.

**Conclusion:**

Our findings suggest a potential role of taurine in both PE pathophysiology and first trimester screening for EO-PE.

## Introduction

Preeclampsia (PE) remains a leading complication of pregnancy and affects approximately 2% of women worldwide [1]–[3]. Early onset PE (EO-PE), requiring delivery before 34 weeks of gestation (GA), is considered the most severe form of the disease in contrast to late onset PE (LO-PE; delivery ≥34 weeks GA) [3]; [4]. Apart from perinatal problems, PE is also associated with substantial health problems later in life. Both women who suffered from PE and their children have a substantially elevated risk of chronic hypertension, cardiovascular disease and diabetes mellitus type-2 [5]. The diagnosis of PE is based on clinical symptoms, such as proteinuria and *de novo* hypertension. These symptoms however are only the terminal features of a cascade of events initiated during the first trimester of pregnancy [6]; [7]. Therefore early recognition of patients at high risk and timely intervention ahead of the clinical onset of the disease would enable suitable pregnancy care and hopefully better pregnancy outcomes for both mother and child.

It becomes more and more clear that PE is caused by interactions between complex pathophysiological mechanisms, individual genes and environmental factors. Some features of the pathophysiology of PE have been elucidated already. Underlying mechanisms comprise impaired early placentation and trophoblast invasion in the spiral arteries, placental hypoxia and endothelial dysfunction [8]–[11]. However, there is currently no complete view on the pathophysiology of PE. Classical approaches to discover novel PE biomarkers are hypothesis-driven and concentrate mostly on the early placental development and maternal adaptation to pregnancy. Other approaches which include novel classes of methods, such as “omics technologies” are “integrated system-based” methods which study general chemical processes in the whole organism to subsequently focus on differences in concentrations of individual molecules, their interactions and role in the pathogenesis.

One of these “omics” technologies is metabolomics. Metabolomics has been successfully applied as biomarker discovery tool for the early detection of diseases such as cancer and cardiovascular disease [12].

Early prediction of PE by using metabolomics analyses on samples derived from maternal blood has been investigated by other groups (Table 1) [13]–[16]. The markers identified differ between studies as do the detection rates, and ranged from 50% in a heterogeneous PE group to 82.6% in an EO-PE group when metabolites are combined with classical PE screening markers such as maternal characteristics, uterine artery Doppler velocity and fetal crown-rump-length (CRL) measurements [13]–[16].

**Table 1:** Studies that assessed preeclampsia screening using metabolomics techniques.

Study	Sample taken (weeks)	Body fluid	Numbers		Gestational age at delivery		Number of metabolites	DR (at FPR)
			Controls	PE	Controls	PE		
Kenny et al [13]								
Discovery study	15±1	plasma	60	60	40.1 (1.1)	37.5 (2.8)	14	77% (10%)
Validation study	15±1	plasma	40	39	38.1 (2.3)	40.0 (1.3)	14	73% (10%)
Odibo et al [14]	12.1 (0.6)	serum	41	41	39.0 (2.8)	35.3 (4.1)	4	50% (10%)
Bahado-Singh et al [15]	11+0–13+6	plasma	60	30	“unaffected”	<34 weeks	4+MC 3+MC+UtA+CRL	75.9% (4.9%) 82.6% (1.6%)
Bahado-Singh et al [16]	11+0–13+6	serum	119	30	“unaffected”	≥37 weeks	2	56.7% (5%)

PE: preeclampsia; DR: detection rate; FPR: false positive rate; MC: maternal characteristics; UtA: Uterine artery Doppler measurement; CRL: crown-rump-length.

In our large nested case-control study we have divided the PE group in two subgroups (EO-PE and LO-PE), as it is known that PE is a heterogeneous syndrome and the pathophysiology of both entities may differ [9]; [17]; [18]. In order to cover the biologically relevant pathways, we selected two metabolomics profiling methods. One has been optimized to target eicosanoids/oxylipins, which are know inflammation markers and the second method targets a wide variety of biogenic amines, including the amino acids.

These groups of metabolites are associated with PE as well other pregnancy complications such as placental abruption, IUGR and preterm birth [19]–[23]. Furthermore both groups are also strongly associated with different inflammatory processes in the cell and endothelial dysfunctions, which are common for PE and cardiovascular disease later in life [24]–[26]. The above associations make them potentially suitable candidate markers for prediction of PE.

The study had two aims: the first aim was to investigate specific signature patterns of metabolites that are significantly altered in first-trimester serum of women who subsequently developed PE compared to healthy pregnancies. The second aim of this study was to examine the predictive performance of the selected metabolites for both EO-PE and LO-PE.

## Material and Methods

### Ethics Statement

This study approach was approved by the Scientific Ethical Committee of the University Medical Centre of Utrecht (METC Utrecht), the Netherlands (protocol number: 11-002). All participating women in this manuscript have given written informed consent during the first-trimester Down syndrome screening.

### Study population

This was a nested case-control study derived from a large cohort of women participating in the routine Dutch first trimester Down syndrome screening between 2007 and 2009. In this context maternal age, sample date, gestational age (GA) at sampling, maternal weight, method of conception, history of diabetes, and smoking status were recorded by a midwife or gynaecologist. As part of the screening, maternal serum concentrations of two standard placental markers (pregnancy-associated plasma protein-A [PAPP-A] and free β-subunit of human Chorionic Gonadotropin [fβ-hCG]) were measured in serum of blood sampled at 8^+0^–13^+6^ weeks GA. After blood withdrawal, samples were centrifuged within 6 hours and stored at 4°C until serum analysis for PAPP-A and fb-hCG. Subsequently, serum samples were stored at −80°C until metabolomics analysis. Thus, samples underwent one freeze-thaw cycle prior to analysis. All samples used in this study were handled the same way. Where applicable, GA was calculated based on first trimester CRL measurement at ultrasound examination using the formula of Robinson and Fleming (1975) [27]; otherwise the first day of the last menstrual period was used. Pregnancy outcomes, including chromosomal disorders, date of birth, birthweight and hypertensive pregnancy complications (PE, HELLP syndrome or pregnancy induced hypertension), were collected through self-reporting by the participating women. Six months after the estimated delivery date, a reminder letter was sent to these women to collect missing data. This way, 75% of all pregnancy outcomes could be recorded. For the current study women with a multiple pregnancy, women who delivered before 24 weeks and women who gave birth to a child with a chromosomal abnormality were excluded.

By follow up of self-reported cases of EO-PE we confirmed the diagnosis of EO-PE in 68 pregnancies at the participating hospitals. Moreover, from a larger cohort of women who developed LO-PE we randomly selected 99 cases. From all EO-PE and LO-PE cases we collected missing data on maternal characteristics i.e. medical history, parity, weight, height, first trimester mean arterial pressure (MAP) and pregnancy outcome i.e. GA at delivery, birthweight and fetal sex. The control group, consisting of 500 women having delivered phenotypically and chromosomally normal neonates at term (37^+0^–42^+0^ weeks) and not having developed any pregnancy complication, was randomly selected from the two largest ultrasound centres participating in the routine Dutch first trimester Down syndrome screening program (Universitair Verloskundig Centrum Utrecht and De Poort Leiden). The outcomes of these pregnancies were confirmed in the midwifery practices and missing maternal characteristics and first trimester MAP were collected.

### Outcome measures

PE was defined according to the criteria of the International Society for the Study of Hypertension in Pregnancy as: gestational hypertension beyond 20 weeks GA in previously normotensive women with a systolic blood pressure ≥140 mm Hg and/or diastolic blood pressure ≥90 mm Hg on at least two occasions four hours apart, with the presence of proteinuria of ≥300 mg in 24-hour collection or at least 2+ by dipstick on a spot urinalysis [6]. Early onset PE (EO-PE) was defined as PE in pregnancies delivering <34 weeks GA, and late onset PE (LO-PE) as PE in pregnancies delivering ≥34 weeks. Pregnancy at term was defined as delivery ≥37 weeks of GA.

MAP was calculated from the formula *DP+1/3 (SP – DP)*, where DP represents diastolic blood pressure and SP -systolic blood pressure.

### Sample analysis

Amine measurements were performed using the method based described previously by Noga et al., 2012 [28]. The amine platform covers amino acids and biogenic amines employing an Accq-tag derivatization strategy adapted from the protocol supplied by Waters (Etten-Leur, The Netherlands). 5 µL of each plasma sample was spiked with an internal standard solution, followed by deproteination by addition of MeOH. The supernatant was transferred to a deactivated autosampler vial (Waters) and dried under N_2_. The residue was reconstituted in borate buffer (pH 8.5) with 6-aminoquinolyl-N-hydroxysuccinimidyl carbamate (AQC) reagent (Waters). After reaction, the vials were transferred to an autosampler tray and cooled to 10°C until the injection (1.0 µL) of the reaction mixture into the UPLC-MS/MS system.

An ACQUITY UPLC system with autosampler (Waters) was coupled online with a Xevo Tandem Quadrupole mass spectrometer (Waters) operated using Masslynx data acquisition software (version 4.1; Waters). The samples were analyzed by UPLC-MS/MS using an Accq-Tag Ultra column (Waters).

The Xevo TQ was used in the positive-ion electrospray mode and all metabolites were monitored in Selective Reaction Monitoring (SRM) using nominal mass resolution.

Acquired data were evaluated using Quanlynx software (Waters), by integration of assigned SRM peaks and normalization using proper internal standards. For analysis of amino acids their ^13^C^15^N-labeled analogs were used. For other amines, the closest-eluting internal standard was employed. Blank samples were used to correct for background, and in-house developed algorithms were applied using the pooled QC samples to compensate for drift in the sensitivity of the mass spectrometer with and over different batches [29].

Measurements of eicosanoids/oxylipins was performed as described earlier by Strassburg et al [30]. After thawing the 250 µL serum aliquots on ice, the samples were treated immediately with antioxidants (0.2 mg 3,5-di-t-butyl-4-hydroxytoluene [BHT]/Ethylenediaminetetraacetic acid [EDTA]) and spiked with a set of 34 isotopically labeled internal standards (ISTDs). Compound extraction was performed with solid phase extraction using Oasis Hydrophilic-lipophilic-balanced reversed-phase sorbent for acids [HLB] (60 mg/30 µm). Oxylipins were eluted with 2 mL ethyl acetate after wetting the cartridge with 0.5 mL methanol. The eluent was reduced under nitrogen. The dried extract was subsequently reconstituted in 50 µL solution of methanol and acetonitrile (1∶1) containing 100 nM 1-cyclohexyluriedo-3-dodecanoic acid (CUDA) as a quality marker for the analysis. Afterwards, the extract was filtered by centrifugation using Amicon Ultrafree-MC Durapore PVDF filter (pore-size 0.1 µM; Millipore, Bedford, MA).

Samples were analyzed by liquid chromatography (LC-MS; Agilent 1260, San Jose, CA, USA) coupled to electrospray ionization on a triple quadrupole mass spectrometer (Agilent 6460, San Jose, CA, USA). For analysis 5 µL of the extract was injected. The auto sampler was cooled at 10°C. Chromatographic separation was achieved on an Ascentis Express (2.1×150 mm, 2.7 µm particles; Sigma-Aldrich Supelco) column using the solvents A, 0.1% acetic acid, and B, 90∶10 *v*/*v* acetonitril/isopropanol. Electrospray ionization was performed in the negative ion mode.

To detect the individual oxylipins, MRM in negative ion mode was performed with individually optimized fragmentor voltage and collision energies (Optimizer application, MassHunter, Agilent). Optimal MRM settings were obtained from flow injection analysis of pure standards using the optimizer application and compared to literature when available. Dynamic MRM was used, assuring optimal dwell time and sufficient data points per peak.

Peak determination and peak area integration was performed automatically with MassHunter Quant (Agilent, Version B.04.00) while auto-integration was manually inspected and corrected if necessary. The obtained peak areas of targets were corrected by appropriate ISTD and calculated response ratios were used throughout the analysis. In-house developed algorithms were applied using the pooled QC samples to compensate for shifts in the sensitivity of the mass spectrometer over different batches [29].

### Statistical analysis

Only metabolites detected in more than 80% of the samples were included in statistical analysis. For other analytes, data below the detection limit, where the LC-MS software provided a missing value, were imputed as half the lowest detectable value for that individual analyte. This replacement was made to get a more realistic value in further calculations and was a compromise between substituting a zero value, which would be an underestimation, and truncating these values at the lowest measurable level, which would be an overestimation of their actual concentrations.

In accordance with the statistical approach described below, the data set was divided into sets for training, testing (evaluation) and validation, respectively. For each group (controls, EO-PE, LO-PE), samples were randomly assigned to the training (40%), test (30%) or validation set (30%). Overall samples assignment was as follows: training set 200 controls, 27 EO-PE, 40 LO-PE; test set 150 controls, 20 EO-PE, 30 LO-PE; validation set 150 controls, 21 EO-PE, 29 LO-PE.

After this random assignment we confirmed that there were no significant differences in maternal characteristics between the three sets (results not shown). The concentration of the (remaining) metabolites and MAP of the training set were expressed as multiples of the gestation-specific normal medians (MoMs). MoMs were log-transformed to provide normal distributions. Normal medians were obtained by regression analysis of the median concentration for each completed gestational week in the controls of the training set, weighted for the number of women tested. Values of MoM were adjusted for variables such as gestation, weight, smoking and ethnicity where MoM values differed significantly between groups (within controls), following the standard methods described by Cuckle and Wald, with curve fitting based on the training set [31].

Maternal characteristics (i.e. medical records, parity, weight and length) were used to calculate prior risks for PE in multiple logistic regression models. The steps undertaken to develop the models for prior risk for EO-PE and LO-PE were described earlier in detail in our recent article [32].

In the next step, metabolites MoM data were compared between controls and either EO-PE or LO-PE, using a Student's t-test. Values were corrected for multiple testing by calculating the False Discovery Rate (FDR).

The potential of MAP and metabolites with FDR<15% as part of a PE prediction model was further tested using logistic regression in R statistical software. Training models were based on training set data (controls and either EO-PE or LO-PE cases) for prior risks and log-MoM data for each significant metabolite as well as all possible metabolites combinations. Models were then tested on test set data for the corresponding metabolites. Models were evaluated based on their predicted Detection Rate [DR] (sensitivity) in the test set for a fixed 10% False Positive Rate [FPR] (1-specificity).

The final models of risk prediction including the metabolite values were calculated in R using separate models for both EO-PE and LO-PE, respectively. The model with the best performance was validated on the validation set.

Since we previously found that PE pregnancies complicated by growth restriction (<10^th^ birth weight centile), had more distinctive classical marker levels that PE alone, both prediction models (EO-PE and LO-PE) were separately examined for both neonates that were appropriate-for-gestational age (AGA) and those who had a birth weight below the 10^th^ centile [32].

Statistical analyses were performed using SPSS (release 20.0; Chicago, IL), SAS software package (release 9.2; SAS Institute, Cary, NC, USA) and R programming language version 2.15 (http://www.r-project.org).

## Results

Baseline characteristics of the study population are shown in *Table 2*. Women who developed PE had higher BMI (EO-PE 24.7, p<0.0001; LO-PE 23.7, p = 0.005), were more often smokers (EO-PE 11.8% vs 4.2%, p = 0.008), and more often had a history of hypertensive pregnancy disorders compared to controls (EO-PE 5.9%, p = 0.009; LO-PE 10.1%, p<0.0001). Furthermore, there were more nulliparous women among the cases (both EO-PE 80.9% and LO-PE 72.7%, p<0.0001).

**Table 2:** Adapted from Kuc et al., 2013 – Study population baseline characteristics in control and PE pregnancies. Values are presented as median (IQR) or number (%) [32].

Characteristics	Controls	EO-PE	LO-PE
	n = 500	n = 68	n = 99
Maternal age (y)	33 (30–35)	34 (30–37)	33 (30–36)
Maternal weight (kg)	65.5 (60.0–73.0)	70.0 (62.0–81.5)*	67.5 (62.0–75.0)
Maternal BMI (kg/m2)	22.8 (20.7–24.8)	24.7 (21.9–29.3)*	23.7 (21.3–26.5)*
Nulliparity	233 (46.6)	55 (80.9)*	72 (72.7)*
Smoking	21 (4.2)	8 (11.8)*	6 (6.1)
Assisted reproduction	0 (0)	3 (4.4)	8 (8.1)
Gestation at sampling (days)	88 (84–91)	85 (76–89)*	85 (79–89)*
History of hypertensive pregnancy disorders	4 (0.8)	4 (5.9)*	10 (10.1)*
Gestation at birth (wk)	40 (39–41)	31 (30–32)*	37 (36–39)*
Birthweight (gr)	3544 (3243–3800)	1300 (1045–1609)*	2650 (2130–3110)*
Birthweight centile	57.0 (33.1–78.4)	25.0 (13.4–50.4)*	13.8 (3.8–46.0)*
Sex, n male (%)	244 (48.8)	34 (49.7)	53 (53.5)

A Pearson's chi square test and Mann-Whitney U test, both with *post hoc* Bonferroni correction were used for statistical analysis. Adjusted significance value p<0.016 (*). EO-PE: early-onset preeclampsia; LO-PE: late-onset preeclampsia; IQR: interquartile range; BMI: body mass index.

### Metabolite pre-selection

Using the data in the training set, we analyzed a total number of 105 potential variables (58 amines, 46 oxylipins and MAP) for statistically significantly different levels between controls and cases of both EO-PE and LO-PE. MAP proved significant for both of these comparisons, with a 10% (MoM ratio case and controls [MoMR] = 1.10) and 7% (MoMR 1.07) increase in EO- and LO-PE, respectively, and both p-values less than 10^−4^ (Table 3). For the amines, taurine and asparagine were significantly different (FDR<15%) between EO-PE and controls, with approximately 20% reduced levels (MoMR 0.79 and 0.84, respectively). Glycylglycine showed a significant reduction in LO-PE of 28%, MoMR 0.72 (Table 3). None of the oxylipins showed a significant (FDR 15%) difference for any group.

**Table 3:** Selection of the markers significantly different between controls and cases (EO-PE or LO-PE) based on training set.

Type marker	Variable	p-value	FDR	MoM ratio case/control
**EO-PE**				
Blood pressure	MAP	<0.0001	<0.0001	1.10
Amine	Taurine	0.0015	0.07	0.79
Amine	Asparagine	0.0043	0.10	0.84
**LO-PE**				
Blood pressure	MAP	<0.0001	<0.0001	1.07
Amine	Glycylglycine	0.0002	0.01	0.72

Student's t-test was used for statistical analysis. Significance value FDR<15%. EO-PE: early-onset preeclampsia; LO-PE: late-onset preeclampsia; MoM: multiple of the median; FDR: false discovery rate; MAP: mean arterial pressure.

### Model selection

Prediction models were fitted based on the training set, using the prior risk and one or more of the significant markers. For EO-PE the model prediction rule was: 1/(1+e∧-(−2.999+33.491 x MAP-MoM −2.490 x Taurine)). For LO-PE the model prediction rule was: 1/(1+e∧-(−1.9792+22.0164 x MAP-MoM −2.490 x Taurine)).

Comparison of the performance of these models on the test set indicated that for EO-PE the highest DR was obtained for the model with prior risk, MAP and taurine. This model gave a DR of 88% at a FPR of 10%, which is a 19% gain on the DR obtained using only the prior risk. We selected this model for further validation. Another model using prior risk, MAP, taurine and asparagine also gave a DR of 88% (Table 4). However, this latter model was not selected, as adding asparagine to the model did not result in further improvement of the DR as compared to the former model.

**Table 4:** Model predicted early preeclampsia detection rate (95% CI) for FPR of 10% with prior risk, MAP, taurine, asparagine and glycylglycine in control and preeclampsia groups.

	Training set		Test set		Validation set	
	DR at 10% FPR (95% CI)	AUC	DR at 10% FPR (95% CI)	AUC	DR at 10% FPR (95% CI)	AUC
**EO-PE**						
Prior risk	30 (16–49)	0.74	69 (48–85)	0.92	30 (14–50)	0.73
Prior risk + MAP	55 (37–72)	0.88	81 (58–92)	0.91		
Prior risk + taurine	48 (31–66)	0.80	65 (43–82)	0.90		
Prior risk + asparagine	36 (22–56)	0.77	70 (48–85)	0.91		
Prior risk + MAP + taurine	55 (37–72)	0.88	88 (70–97)*	0.93	55 (36–76)	0.78
Prior risk + MAP + asparagine	55(37–72)	0.87	75 (53–89)	0.91		
Prior risk+ MAP + taurine + asparagine	55 (37–72)	0.87	88 (70–97)	0.93		
**LO-PE**						
Prior risk	37 (24–53)	0.75	38 (22–55)	0.70	17 (8–35)	0.55
Prior risk + MAP	43 (28–58)	0.81	46 (30–64)*	0.79	17 (8–35)	0.65
Prior risk + glycylglycine	46 (31–60)	0.79	38 (21–59)	0.72		
Prior risk + MAP + glycylglycine	53 (37–67)	0.83	42 (27–61)	0.78		

DR: detection rate; FPR: false positive rate; MAP: Mean Arterial Pressure; CI: confidence interval; AUC: area under curve, EO-PE: early-onset preeclampsia; LO-PE: late-onset preeclampsia.

*The best model selected for further validation.

For LO-PE, the best predicting model included prior risk and MAP. This model did not include any metabolites (Table 4).

### Validation

Applying the selected models to independent data validated the EO-PE model. This model gave a DR of 55%, which was a 25% improvement to the DR obtained using only the prior risk (Table 4). This gain of 25% was also obtained for the training set.

**Figure 1:**
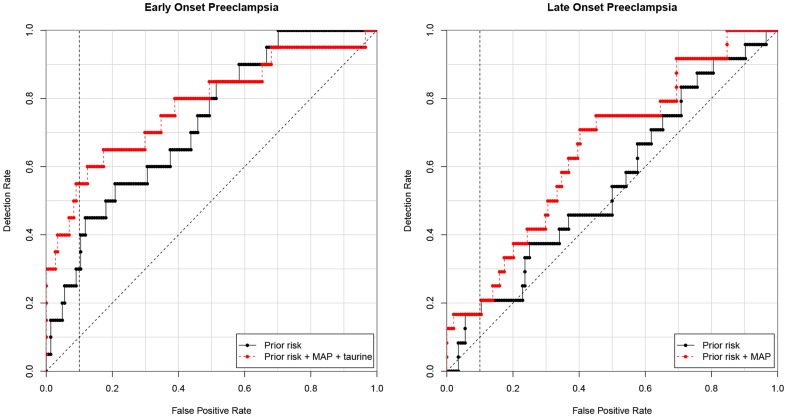
Receiver operating characteristic curves (ROCs) with prediction models for early onset preeclampsia and late onset preeclampsia used on the validation set. Prior risk of preeclampsia containing maternal characteristics (black line) and prior risk for preeclampsia combined with different markers (red line). MAP: mean arterial pressure.

The LO-PE model resulted in a DR of 17% (Table 4). Neither metabolic marker nor MAP improved this model. Final models for both EO-PE and LO-PE are shown in Figure 1. Additionally both models (EO-PE and LO-PE) were separately examined for growth-restricted fetuses. None of the metabolites showed any improvement in DR in these particular subgroups.

## Discussion

Early detection of PE would allow the development and application of targeted intervention. Detection, however, has been restricted to clinical parameters such as high blood pressure and presence of protein in the urine until recently. When these parameters are present, the condition has already progressed. An assay-detecting women at risk to develop PE at an early stage would therefore be extremely helpful in preventing its severe symptoms at the end of the pregnancy. To be able to develop such an effective first trimester prenatal screening test, sets of biomarkers are needed that are capable of detecting the presence of pathologic conditions in maternal serum with high sensitivity and specificity.

In this study we applied LC-MS based metabolomics to assess the diagnostic potency of metabolites such as amines and oxylipins to predict EO-PE en LO-PE from first trimester maternal serum. Using standardized metabolomics analytical techniques and an established statistical approach we identified two molecules of which the serum concentrations were significantly decreased in the first trimester maternal serum of women who subsequently develop EO-PE, and one metabolite with decreased levels in LO-PE compared to controls. All three compounds (taurine, asparagine and glycylglycine) are amino acids or amino acid derivates. Altered levels of amino acids in maternal fluids have earlier been observed in preeclampsia and fetal growth restriction [33]–[37]. Interestingly, they reported a clear association of the properties of taurine and glycylglycine and the possible pathogenesis of PE. In contrast to previous studies performed in the second and third trimester of pregnancy we did not find any differences in eicosanoids and oxylipins between PE and controls. However, PE is a dynamic process, and the metabolic expression profiles may change during different stages of pregnancy which might explain why we could not confirm up- or downregulation of any of these metabolites.

Taurine is a sulfur-containing amino acid-like endogenous compound found in substantial amounts in mammalian tissues. Among its many functions, taurine is an important regulator of antioxidation and membrane stabilization [38]. It is the most abundant free amino acid –derivative in human placenta [39]–[40]. Through its cytoprotective role and as a regulator of cell volume, taurine is thought to be involved in placental trophoblast development, during the remodeling of the spiral arteries early in the pregnancy [40]. Reduced activity of taurine transporters in the placental trophoblast with shortage of taurine in placental tissue is strongly associated with impaired trophoblast invasion into the spiral arteries [40]. Impaired placental trophoblast invasion is one of the most important components of PE pathology [9]; [17]. Furthermore, taurine has also been associated with cardio- and vasoprotective effects through the influence of renin-angiotensin-aldosterone system [38]. From different animal model studies taurine appears to have antihypertensive effects and to reduce total peripheral vessel resistance. Spontaneously hypertensive rats supplemented with taurine show dosage dependant blood pressure reduction [41]. Taurine supplementation during pregnancy in rats leads to reduction of hypertension in their offspring as well [41]–[43].

Glycylglycine is a dipeptide of glycine. The latter one appears to be an essential amino acid in pregnancy and fetal cardiovascular development since its supplementation prevents elevation of blood pressure in rats [44]. Furthermore, glycine is an important component of the S-amino acid metabolic pathway as it antagonises homocysteine levels [45]; [46]. Through the biological feedback loop, lower levels of glycine lead to higher levels of homocysteine. Hyperhomocysteinemia is a known risk factor for endothelial dysfunction, PE and, also cardiovascular disease [47]–[50].

So far, this is the first study associating lower levels of asparagine to PE or any other aspect of cardiovascular disease. Although the role of asparagine in PE development is not yet known, our results may suggest that the shortage of asparagine may be a risk factor for development of EO-PE.

All three metabolites are very interesting concerning the pathophysiology of PE. However, there is still very little known about the insights of the underlying mechanisms and possible underlying cause of PE. Due to technical and financial restrictions we only used two metabolomics platforms (amines and eicosanoids/oxylipins) in this study. These two groups are strongly associated with inflammatory processes in the cell and with endothelial dysfunctions, which are commonly present in PE pregnancies [19]–[26] and therefore likely to reveal relevant biomarkers. Nevertheless, there are many pathways associated with the pathogenesis of PE, that would not be reflected here, so future studies might also explore other metabolic profiles.

None of the significant metabolites that we found have been brought to light by previous metabolomics studies [13]–[16]. From all 105 metabolites we tested, five were reported to be important in previous studies (methylhistidine, alanine, phenylalanine, methionine and valine). However, in our study these five did not differ between PE and control groups. There are hardly matches in significant metabolites selected by different groups [13]–[16]. This strengthens the assumption of the major complexity of the syndrome and the origins of its pathology. The causative complex of interacting mechanistically blameworthy factors as well as the likely presence of confounding factors in a system that inherently changes over time, may therefore lead to differences not only between two variants of the disease (EO-PE vs LO-PE), but also to differences between subsets of patient and control cohorts and even from one study to the next. Reproducibility of results is often difficult in biomarker studies. Other metabolomics profiling studies, on cancer for example, face similar difficulties [51]–[52]. This is mostly due to the fact that samples are taken or manufactured at different conditions, at different time moments, or in different populations. This is a shortcoming and a limitation of this particular method. Therefore, further evaluation of the metabolites identified in our study should be performed in different populations.

The prediction model using a combination of prior risk with MAP and taurine provided a DR of 55% at a fixed 10% FPR in the case of EO-PE, which is comparable to the results, obtained in training. Addition of asparagine did not further improve the results (Table 4). For LO-PE a model using prior risk performed best and it gave a DR of 15% on the validation set. Addition of MAP did not improve the DR, however the AUC increased. Glycylglycine, although significantly decreased in LO-PE versus controls (Table 3), did not turn out to be sufficiently additionally informative to become included in the final prediction model. Therefore, taurine remains the single predictive metabolite marker of this study. Because of its narrow affiliation with placentation, hypertension and cardiovascular disease, a potential screening role of taurine is conceivable.

Ideally, a biomarker for PE should be predictive throughout the entire first trimester. However, due to different processes that are involved in early pregnancy and placentation other biomarkers might be predictive at different gestational weeks. Most of our samples were taken after 11 weeks of gestation; a subanalysis of our data indicated that the metabolites identified in this study were still found in samples from 11 weeks onwards only (data not shown).

We chose not to combine the metabolites with classical first-trimester screening markers such as Pregnancy Associated Plasma Protein-A (PAPP-A), A Disintegrin And Metalloprotease 12 (ADAM12), Placental Protein 13 (PP13), Placental Growth Factor (PlGF). Given our results, it is plausible that the DR of PE would increase if taurine would be added, although the added value in such a large-scale screening setting, also in relation to increased costs and logistics, would need to be established in a larger population before further recommendations can be made. Until such validation studies have been carried out our results should be considered preliminary.

In conclusion, three markers out of 105 were significantly different between women who developed PE and healthy individuals, but after adequate statistical analysis only taurine remained as a predictive marker in the screening model. However, given the possible role of taurine in hypertension treatment, its role as a possible screening marker or maybe even as a diet supplement early in pregnancy remains very interesting.
