# Correction: Metabolomics Profiling for Identification of Novel Potential Markers in Early Prediction of Preeclampsia

**DOI:** 10.1371/journal.pone.0106358

**Published:** 2014-08-19

**Authors:** The PLOS ONE Staff

There are errors in Table 1. The corrected table is shown below.

**Table 1:** Studies that assessed preeclampsia screening using metabolomics techniques. PE: preeclampsia; DR: detection rate; FPR: false positive rate; MC: maternal characteristics; UtA: Uterine artery Doppler measurement; CRL: crown- rump-length.

Study	Sample taken (weeks)	Body fluid	Numbers		Gestational age at delivery		Number of metabolites	DR (at FPR)
			Controls	PE	Controls	PE		
Kenny et al [13] *Discovery study *	15 ± 1	plasma	60	60	40.1 (1.1)	37.5 (2.8)	14	77% (10%)
*Validation study*	15 ± 1	plasma	40	39	38.1 (2.3)	40.0 (1.3)	14	73% (10%)
Odibo et al [14]	12.1 (0.6)	serum	41	41	39.0 (2.8)	35.3 (4.1)	4	50% (10%)
Bahado-Singh et al [15]	11+0 – 13+6	plasma	60	30	“unaffected”	< 34 weeks	4 + MC	75.9% (4.9%)
							3 + MC + UtA + CRL	82.6% (1.6%)
Bahado-Singh et al [16]	11+0 – 13+6	serum	119	30	“unaffected”	≥ 37 weeks	2	56.7% (5%)
